# A rare case of intramedullary ‘whorling-sclerosing’ variant meningioma

**DOI:** 10.1186/s40064-015-1110-8

**Published:** 2015-07-04

**Authors:** Ghazala Perven, Pouya Entezami, Daniel Gaudin

**Affiliations:** Department of Neurology, University of Toledo Medical Center, Toledo, OH USA; Department of Surgery/Division of Neurosurgery, University of Toledo Medical Center, 3000 Arlington Avenue, Toledo, OH 43614 USA

**Keywords:** Meningioma, Intramedullary tumor, Whorling-sclerosing

## Abstract

A 52-year-old man with a seven-year history of progressive weakness, gait problems, and pain in his extremities presented with subacute worsening of his symptoms. Examination revealed weakness in all four extremities, increased tone, hyperreflexia, and sensory deficits. MRI of the cervical spine showed an area of signal abnormality and abnormal enhancement within the cervical cord at the C5–C6 level. The patient initially underwent biopsy followed a few days later by a debulking surgery. Postoperatively, the patient showed improvement in strength as well as ambulation. Intraoperatively, the lesion was confirmed to be intramedullary without any dural attachments. Histopathological examination revealed an extensively hyalinized tumor with sparse collections of cells that were immunopositive for both cytokeratin and GFAP, and immunonegative for EMA and progesterone receptor. This is an unusual pattern of expression, with cytokeratin immunopositivity suggesting a meningioma and GFAP immunopositivity suggesting a glioma. Considering the combination of extensive hyalinization with cytokeratin positivity the tumor was thought to be most consistent with a hyalinized meningioma with GFAP positivity. GFAP-positive meningiomas are rare, and these include the recently described ‘whorling-sclerosing’ variant. Only three cases of this tumor have been previously reported, all of which were intracranial. This is the first reported case of an intramedullary whorling-sclerosing meningioma.

## Background

Meningiomas are generally benign tumors originating from non-neuroepithelial progenitor cells, known as arachnoid cap cells. The WHO classification identifies fifteen distinct histological variants (Kleihues et al. 2002) but other variants exist as well. Intraspinal meningiomas are relatively frequent primary tumors of the spinal cord. Approximately 25% of all primary spinal cord tumors are meningiomas (Chamberlain and Tredway 2011).

Meningiomas found within the spinal canal most commonly affect the thoracic region (80%), though cervical (15%) and lumbosacral (5%) tumors are also observed (Van Goethem et al. 2004). These tumors are most commonly intradural and extramedullary, though there are a few reports in the literature of intramedullary meningiomas.

We present a 52-year-old man with a whorling-sclerosing variant meningioma of the spine. Only three cases with this histological pattern have previously been reported, and all of these were intracranial (Pope et al. 2003; Haberler et al. 2002). We present the first reported spinal tumor with this histological presentation.

## Case report

A 52-year-old man with a seven-year history of progressive weakness, gait problems, and pain in his extremities presented with subacute worsening of his symptoms. Initially, he was hesitant about undergoing surgery and was managed conservatively, but his weakness continued to progress and he had become wheelchair-bound. Examination revealed weakness in all four extremities, increased tone, hyperreflexia, and sensory deficits. Bowel and bladder functions were spared. The patient received corticosteroids that helped alleviate some of his symptoms but, due to the progressive course, the patient consented for surgery.

**Figure 1:**
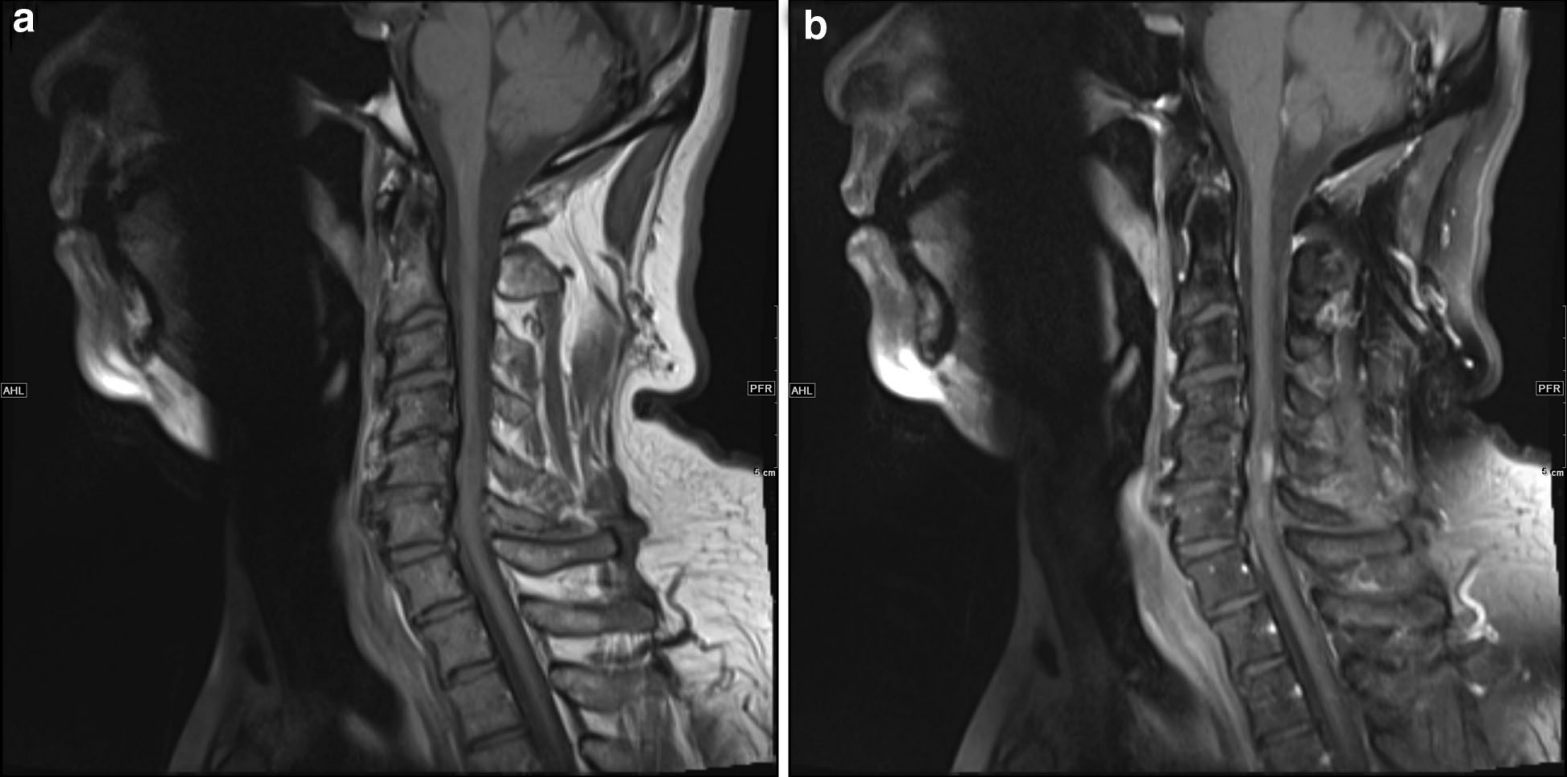
Sagittal T1 MRI (**a**) of the cervical spinal cord shows an area of mixed signal intensity as well as slight expansion at C4–C5 and C5–C6. Post-contrast images (**b**) show an area of enhancement within the cord at the level of C4–C5.

**Figure 2:**
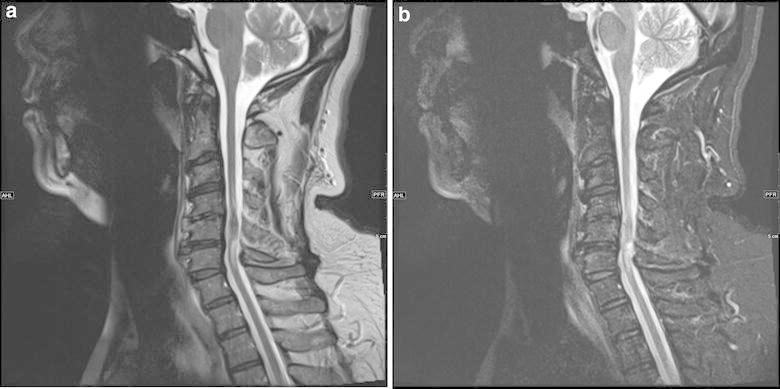
Sagittal T2 MRI (**a**) and sagittal STIR (**b**) of the cervical spinal cord shows heterogeneous signal intensity with surrounding edema at C4–C5.

MRI of the cervical spine showed an area of signal abnormality as well as abnormal enhancement within the cervical cord at the C5–C6 level. The lesion was hypointense on T1 (Figure 1) and hyperintense on T2 sequence (Figure 2) extending one vertebral level up and one vertebral level down. The cord was noted to be expanded at the level of the lesion.

**Figure 3:**
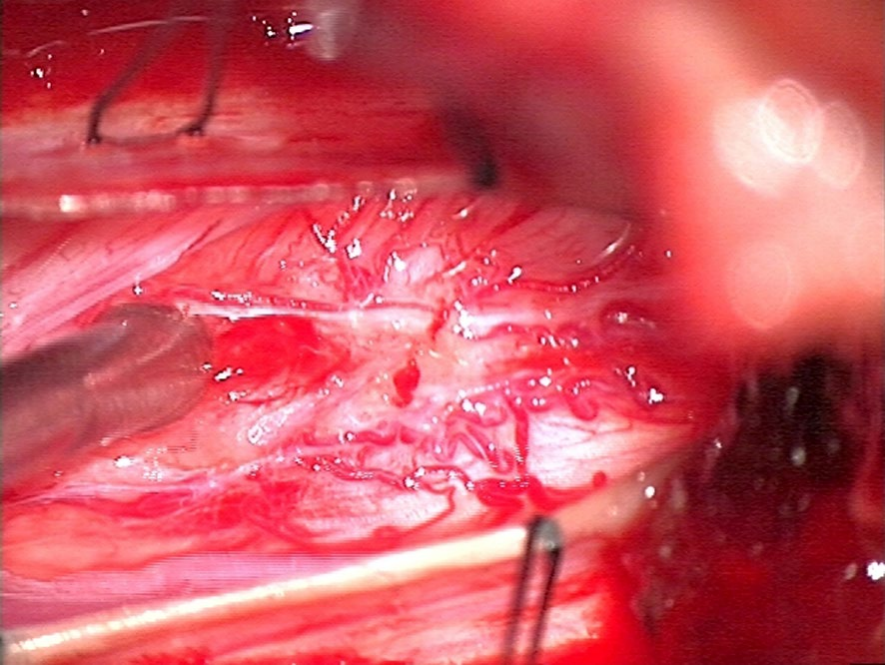
Intraoperative appearance of the tumor after myelotomy.

The patient underwent a biopsy, via C4–C5 laminectomy and C6 hemilaminectomy. Midline dissection of the cord at C5 revealed a tan-grey tumor, which was biopsied. One week postoperatively patient underwent debulking surgery under ultrasound guidance and a tumor with dimensions of 1.1 cm × 4 mm × 4 mm was removed using microsurgical technique (Figure 3).

Postoperatively, the patient showed improvement in strength as well as ambulation. By 6 months he was able to ambulate using a walker for short distances. He also reported significant improvement in pain and paresthesias in his arms and legs, though he continued to have residual neurological deficits, including a right hand contracture.

**Figure 4:**
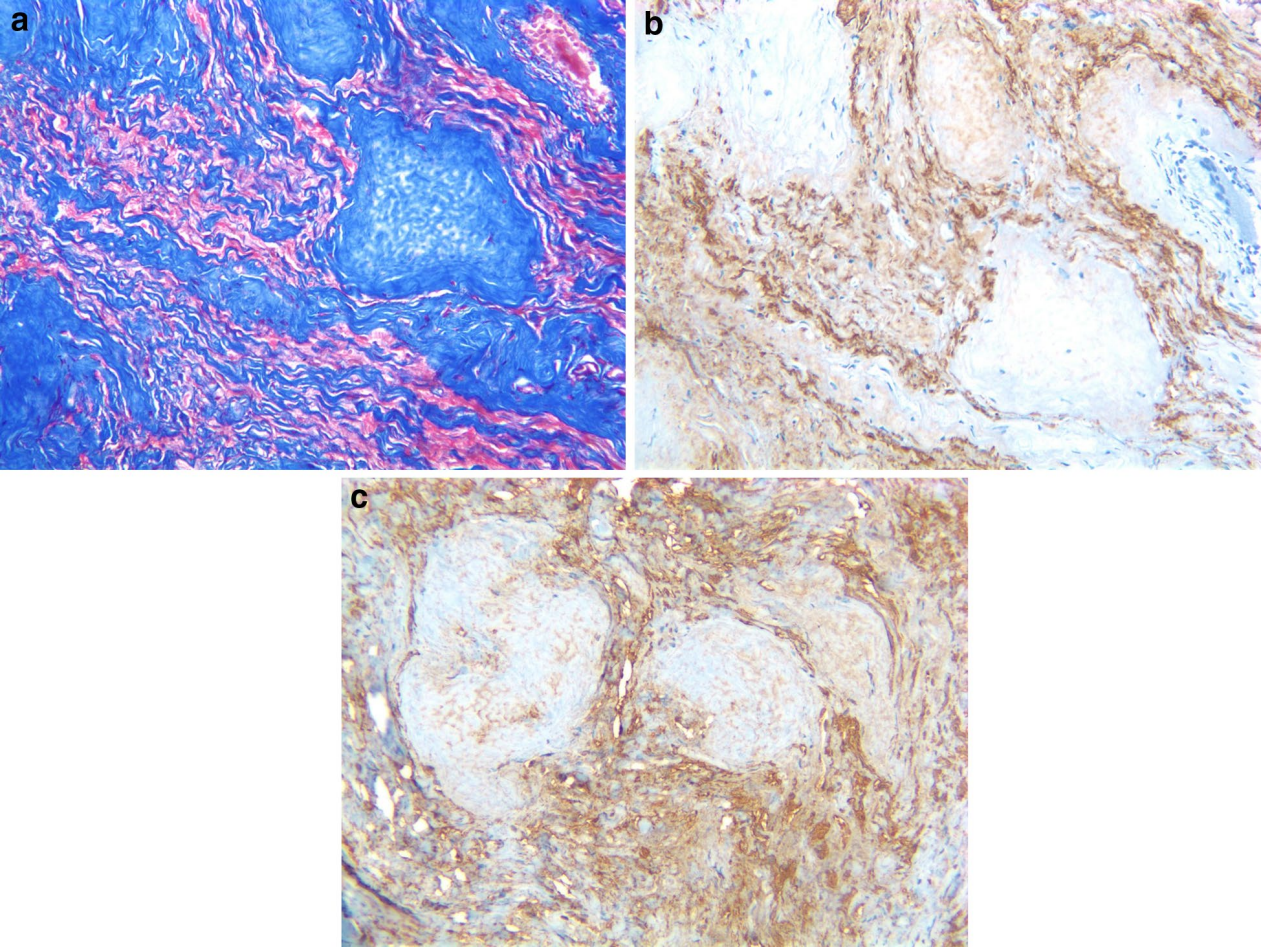
Histological appearance with **a** trichrome stain; **b** immunopositive for cytokeratin; **c** immunopositive for GFAP.

Histopathological examination revealed an extensively hyalinized tumor with sparse collections of cells. The collagenous nature of the hyalinized material was confirmed with a trichrome stain (Figure 4a). The benign nature of the tumor was suggested by the bland nature of the cellular component and was confirmed with a KI-67 immunoreaction, which showed almost no proliferating cells. A PAS stain showed no eosinophilic granular bodies. The cells were immunopositive for both cytokeratin (Figure 4b) and GFAP (Figure 4c), and immunonegative for epithelial membrane antigen (EMA) and progesterone receptor.

## Discussion

We present the first reported case of an intraspinal, GFAP-positive meningioma. Our case is similar to two cases described by Haberler et al. (2002) and one case described by Pope et al. (2003) in both histological and immunological findings. These authors suggested that this particular histological pattern be named the ‘whorling-sclerosing’ variant of meningioma. There are a number of examples of GFAP-positive meningioma reported in literature but these all are intracranial and extra-axial (Table 1).

**Table 1:** GFAP-positive meningiomas; review of cases in the literature

References	Age/gender	Location	Histological diagnosis	Immunohistochemistry	
				Positive	Negative
Budka (1986)	48 years/F	Extra-axial left parietal	Papillary meningioma	GFAP, vimentin, cytokeratin	
Wanschitz et al. (1995)	24 years/F	Suprasellar	Chordoid or papillary meningioma	GFAP, NSE, S100, vimentin, cytokeratin, and EMA	SYN, NFP, CHROM A, CEA, FN, desmin, MU 128-UC
Su et al. (1997)	63 years/M	Extra-axial in the right superior and medial frontal gyri	Atypical meningioma: meningiothelial type	EMA, vimentin, GFAP	S 100 protein
Haberler et al. (2002)	48 years/F	Bifronto-basal invading skull base, sinuses and orbit	Whorling-sclerosing variant of meningioma	S100 protein, vimentin, EMA, CD34, GFAP	Cytokeratin, progesterone, desmin
Haberler et al. (2002)	77 years/M	Dural based right occipital	Whorling-sclerosing variant of meningioma	EMA, cytokeratin, desmin, S100, vimentin, GFAP	CD34, pancytokeratin, progesterone
Pope et al. (2003)	34 years/M	Dura of mesencephalon and pons	Whorling-sclerosing variant of meningioma	EMA, vimentin, GFAP	Cytokeratin, CEA

*GFAP* glial fibrillary acidic protein, *NSE* neuron specific enolase, *EMA* epithelial membrane antigen, *SYN* synaptophysin, *NFP* neurofilament protein, *CHROM A* chromogranin A, *FN* fibronectin, *CEA* carciniembryonic antigen, *MU 128-UC* smooth muscle actin.

Glial fibrillary acidic protein (GFAP) is specific for neuroglial filaments and has been used as a reliable marker for normal, reactive, and neoplastic astrocytes. It is a structural protein which is the predominant component of glial intermediate filaments. Therefore, a tissue immunopositive for GFAP is assumed to be of glial origin. More recently GFAP immunopositivity has been recognized in other types of neoplastic and normal tissues as well, including epiglottic cartilage, renal carcinoma metastatic to brain, malignant pleomorphic adenoma of the salivary glands, and papillary meningioma (Budka 1986).

Our case also represents a rare example of cervical intramedullary meningioma. Completely intramedullary meningiomas in the spinal cord are rare, with only six reported cases in the literature (Table 2). Five of these tumors were located in the cervical area with one extending up to the cervicomedullary junction. This is in contrast to extramedullary meningiomas, which are preferentially located in thoracic segments of the cord. The histopathological appearance of the six reported intramedullary meningiomas was variable, with two being clear cell meningiomas (Park et al. 2006; Jallo et al. 2001), one a transitional meningioma (Moriuchi et al. 1996), one an atypical grade II meningioma (Sahni et al. 2008), one a fibroblastic meningioma (Salvati et al. 2007), and one a syncytial-type meningioma (Salehpour et al. 1992). This is the first reported case of an intramedullary spinal cord whorling-sclerosing meningioma.

**Table 2:** Intramedullary spinal cord meningiomas; review of cases in literature

References	Age/gender	Location	Histology
Moriuchi et al. (1996)	54 years/F	C2–C4	Transitional meningioma
Park et al. (2006)	65 years/F	T9–T10	Clear cell meningioma
Sahni et al. (2008)	42 years/M	C3–T2	Atypical meningioma (WHO grade 2)
Salvati et al. (1992)	67 years/F	C2–C4	Fibroblastic meningioma
Salehpour et al. (2008)	21 years/M	Cervicomedullary junction–C2	Syncytial type meningioma
Jallo et al. (2001)	22 months/F	C3–C5	Clear cell meningioma

## Conclusion

Our report confirms previous reports that spinal meningiomas can be intramedullary and that GFAP positivity is not specific for glial tumors. Fortunately, management is unchanged for these variants and surgical resection carries a good prognosis. Nevertheless, the ‘whorling-sclerosing’ variant of meningioma is a rare type of neoplasm with a specific histopathological and immunological profile that should be recognized.

## Consent

Patient consent has been obtained and is on-file at the University of Toledo Medical Center, Toledo, OH, USA.
